# UAV-Based Smart Parking Occupancy Detection and Management Using YOLOv11x-OBB

**DOI:** 10.3390/s26216940

**Published:** 2026-11-02

**Authors:** Sek Joo Shan, Mazen Farid

**Affiliations:** 1 Faculty of Information Science and Technology (FIST), Multimedia University, Melaka 75450, Malaysia; 2 Centre for Intelligent Cloud Computing, COE for Advanced Cloud, Multimedia University, Melaka 75450, Malaysia

**Keywords:** computer vision, drone, YOLO oriented bounding box, parking slot tracking, flask, real-time web application

## Abstract

With the rapid growth of motor vehicle ownership across cities, parking lots attached to large commercial supermarkets and transportation hubs generally face the common pain points of delayed vacant parking spot scheduling and low manual inspection efficiency. The market urgently needs an intelligent parking management system that can quickly cover an entire site and report real-time updates of parking space status. To address this gap, this paper presents an integrated system for visual parking occupancy detection based on drone aerial imagery, combining an oriented-bounding-box vehicle detector, automatic parking-slot discovery and tracking, and a dual-role web platform for managers and customers. Rather than proposing a new detection algorithm, the contribution of this work lies in the automatic, annotation-free slot inference, tracking and gap-filling mechanism and in the end-to-end system integration that removes the need for manual slot mapping or per-site calibration. The system was field tested on three real-world parking lots of increasing size and density and achieved an occupancy-classification accuracy of 82.4% and F1 of 0.805 (detection F1 = 0.827 and mAP@0.5 = 0.839) against manually annotated ground truth on 51 annotated frames, with a slot-discovery coverage (Slot Recall) of 0.833; these independently evaluated figures are prioritised over, as well as considerably lower than, the system’s own temporal-consistency detection scores of 98.7% accuracy/98.8% F1, which measures prediction stability without manual annotation. An itemised cost breakdown for a representative 10,000 m^2^ (~112-bay) lot indicates a deployment cost of RM6409 for the camera/UAV-based approach versus RM29,973 for an equivalent geomagnetic-sensor solution, which is approximately 21.4% of the geomagnetic up-front total (purchase and installation only); recurring UAV operating costs for continuous monitoring are discussed separately. The proposed approach offers a low-cost, sensor-free path toward intelligent parking management for large-scale lots.

## 1. Introduction

With the acceleration of global urbanisation, the number of private cars in cities has continued to increase. The process of drivers searching for parking lots often causes additional traffic congestion, rising fuel consumption, and increased carbon emissions, which lowers the overall operational efficiency of urban transportation. For this reason, smart parking management has become an indispensable core component of modern urban transportation systems.

Currently, traditional parking monitoring solutions mainly fall into two categories. The first category uses fixed cameras, and the second category relies on geomagnetic or ultrasonic sensors. By applying this approach, accurate spot-level parking detection can be achieved and provide a stable monitoring perspective. However, it suffers from limited coverage, severe scene occlusion problems, and excessively high deployment and later maintenance costs.

However, this approach requires each parking spot to be fitted with an individual sensor, which not only leads to high deployment costs but also has poor scalability in large parking lots or temporary parking scenarios. In recent years, the rapid development of computer vision, artificial intelligence, and unmanned aerial vehicle (UAV) technology has created a new implementation path for smart parking monitoring.

Compared with traditional solutions, UAV-based aerial monitoring offers wider coverage, a lower chance of being blocked by obstructions, and more flexible deployment. However, it also brings two main visual analysis challenges: accurate detection of vehicles oriented in any direction and reliable determination of the occupancy status of each individual parking slots.

To address these issues, this study proposes a UAV-mounted real-time parking occupancy detection system based on computer vision technology. The system can process aerial video streams to automatically identify vehicles and determine parking slots’ status, aiming to improve parking management efficiency, reduce traffic congestion caused by parking search, and support the construction of smart urban transportation systems.

The proposed system has been validated on real-world parking lot datasets from three sites, which have small, medium and large parking spots. It achieves an occupancy-classification accuracy of 82.4% and an F1-score of 0.805 against the manually annotated ground truth (the internal temporal-consistency scores of 98.7% accuracy/98.8% F1 measure prediction stability and are considerably more optimistic) and requires no additional calibration for specific parking lots. The scheme proposed in this study, which integrates UAV-based aerial monitoring and computer vision, is a low-cost, sensor-free approach to intelligent parking management; the broader scalability across sites, altitudes and conditions is discussed as a direction for future validation in Section 7.

The following Section 2 reviews related work on vision-based object detection for drones and parking monitoring and identifies the research gap that motivates the proposed system. Section 3 formalizes the methodology, including the detection model, oriented-bounding-box geometry, intersection-over-union occupancy criterion and temporal-consistency quality metrics. Section 4 presents the proposed system design and its dual-role web architecture. Section 5 details the implementation of the detection engine, slot-tracker and streaming pipeline. Section 6 reports and analyses the experimental results on three aerial parking lots, and Section 7 concludes the paper and outlines directions for future work.

## 2. Related Work

Object detectors, such as YOLOv8, SSD MobileNet-V2, have been used on drones for navigation and landing: Tullu et al. [1] paired YOLOv3 with a lightweight planner for real-time 3D path planning; Godase et al. [2] optimised embedded YOLOv8 variants for A*/RRT* obstacle avoidance; and Safadinho et al. [3] used SSD/MobileNet-V2 to detect a human receiver for marker-free landing-site selection.

While these detectors establish the maturity of vision-based detection on drones, they target flight tasks rather than ground-based parking monitoring. A separate body of work addresses smart parking and automatic parking-slot detection directly. Peng et al. [4] demonstrated drone-based vacant parking-space detection using classical image processing on aerial footage, establishing the early feasibility of overhead drone monitoring for parking occupancy but relying on fixed, manually surveyed slot positions. Grbić and Koch [5] proposed an automatic vision-based parking-slot detection and occupancy classification method that infers slot positions from clustered vehicle detections over a sequence of images rather than requiring manual annotation, closely paralleling the slot-discovery goal of the present system, though their method targets fixed ground-level or elevated cameras rather than a mobile UAV platform. More recently, YOLOv11-based detectors have been applied directly to parking-space detection: Aguswandi et al. [6] trained a YOLOv11 model for empty-slot detection, achieving high reported precision, recall and mAP with real-time inference around 25 FPS, though again from a fixed camera viewpoint rather than aerial UAV footage and without automatic slot discovery or occupancy tracking over time.

Traditional smart parking systems rely on per-bay physical sensors, such as ultrasonic and magnetic devices, or on entrance/exit counters. These systems provide reliable local detection but require hardware installation and maintenance at every bay, and counter-based approaches cannot identify the location of individual vacant spaces, which limits their usefulness for space-level parking guidance.

Computer vision provides an alternative by using fixed surveillance cameras to monitor multiple parking spaces simultaneously. Early methods employ background subtraction, Canny edge detection, local binary pattern (LBP) features, and perceptual hashing to distinguish occupied spaces from vacant ones while reducing the effects of lighting variations and pedestrian movement [7]. Other systems analyse pixel-intensity changes across video frames to estimate parking occupancy and provide web-based parking information [8]. However, fixed-camera systems remain limited by restricted viewing angles, vehicle occlusion, blind spots, and the need for permanent camera installation.

Unmanned aerial vehicles (UAVs) offer flexible, infrastructure-independent monitoring by capturing aerial views of extensive parking areas. Early approaches use scale-invariant feature transform (SIFT) descriptors and K-means clustering to estimate vehicle density, reducing reliance on manually annotated bounding boxes [9]. More advanced frameworks coordinate multiple drones using route-planning algorithms, such as the Vehicle Routing Problem (VRP) and Travelling Salesman Problem (TSP), combined with CNN-based vehicle analysis to inspect parking areas and identify improperly parked vehicles [10].

More recently, single-stage object detectors from the YOLO family have become widely used for real-time vehicle detection in aerial imagery. Models such as YOLOv5, YOLOv8, and YOLOv11 support vehicle localisation and occupancy analysis [11], and lightweight multi-scale detectors with geometry-aware regression have been proposed to improve the localisation of small, densely packed vehicles and rectangular parking spaces in UAV images [12].

Emerging approaches also explore vision-language models (VLMs) for parking analysis: DroneVision (Keelung City, Taiwan) uses a VLM to analyse drone imagery and generate occupancy reports without retraining a task-specific model [13].

Despite these developments, challenges remain in combining flexible aerial monitoring, accurate parking-slot localisation, stable occupancy classification, and real-time operation within a practical parking-management system. This study addresses these challenges using YOLOv11x-OBB to analyse UAV-captured parking videos and detect vehicle locations for slot-occupancy classification. The system also incorporates slot tracking and automatic slot-layout completion, with a manager interface for monitoring results and an SVG-based customer map for viewing parking availability.

Vision-based detectors on drones have mostly been explored for flight activities such as obstacle avoidance and landing [1,2,3]. Not many researchers explore the potential of overhead imaging from drones in structured monitoring scenarios on the ground. Those who have done so often need manual slot mapping or per-site calibration. Building on the automatic slot-detection approach of Grbić and Koch [5] and the aerial YOLO-based detection demonstrated in [11,12], the current effort combines an oriented-bounding-box detector with temporal tracking and gap-filling to achieve automatic slot discovery and real-time occupancy estimation from a UAV viewpoint, without per-site calibration or manually labelled slot maps. A quantitative comparison against the published video-based YOLOv5 parking-occupancy baseline of Ogawa et al. [14], using their own reported metrics, is provided in Section 6, together with an ablation study isolating the contribution of the oriented-box detector, temporal filtering and gap-filling.

Within this body of work (Table 1), parking-specific studies remain comparatively few: most rely on fixed ground-level cameras or per-bay sensors rather than UAV-mounted imagery, require manual slot annotation that does not transfer across sites, or, like Grbić and Koch [5] above, report slot detection and occupancy classification without an integrated tracking, gap-filling or deployment-ready web interface. The present system is positioned to close these specific gaps rather than to extend general UAV flight autonomy; Section 6.5 benchmarks quantitatively against a published video-based baseline by Ogawa et al. [14].

## 3. Methodology

### 3.1. Hardware Platform

The framework has been implemented using a standard Windows 11 laptop equipped with an Intel Core i7-12700H processor, 16 GB RAM, and an NVIDIA GeForce RTX 3060 GPU (6 GB VRAM). Under continuous operation, the system sustained a mean of 26.3 FPS, with a mean end-to-end latency of 38 ms per frame (frame capture and resize: 5 ms; YOLOv11x-OBB inference: 28 ms; slot tracking and occupancy logic: 3 ms; and JPEG encoding and web serving: 2 ms). Without a GPU, the framework falls back on the CPU, which sustains approximately 2.1 FPS (around 480 ms per frame on the same i7-12700H). As for the aerial video stream, it is captured using a DJI Flip (SZ DJI Technology Co., Ltd., Shenzhen, China) quadcopter hovering at a height of 30 to 50 m, with its camera lens directed straight down, broadcasting H.264 video streams to the ground station through the RTMP protocol using the DJI Flip application.

Even though the experiments process pre-recorded and live feeds from drones at the ground station, the pipeline itself does not depend on hardware and accepts any OpenCV 5.0.0-compatible feed: USB webcams, RTSP/RTMP streaming, video files and images via the same interface. In the case of the DJI Flip, the H.264 video feed over WiFi is streamed to an nginx-rtmp relay server, from which individual frames can be grabbed on demand. This decoupling of video capture from the processing engine allows for discarding old frames in the case of a high load and operating only on the latest frame available, which is important for real-time occupancy monitoring.

### 3.2. Software Stack

This system was developed using Python 3.11 and has an associated frontend written in JavaScript. The system uses the Ultralytics YOLOv11x-OBB with the pre-trained DOTAv1 weights, which is capable of detecting the aerial vehicles at arbitrary orientations. OpenCV (cv2 ≥ 4.8) captures the frame, scales, calculates the polygons’ IOU values, and encodes the result as a JPEG image. All coordinate calculations and time-stamped history arrays depend on the NumPy library. Flask (≥3.0) provides the web interface with role-based sessions and an implementation of a multi-part MJPEG Response object generator. Three web pages are rendered by Jinja2, and the customer and manager parking map interfaces were created with Vanilla JavaScript and HTML5 Canvas/SVG. Parking slots are saved between sessions using a Python pickle file (slots.pkl). The drone’s live feed is captured from an nginx-rtmp server on port 1935.

Each element is responsible for a specific purpose. The YOLOv11x-OBB model and inference API are provided by Ultralytics (Frederick, MD, USA), OpenCV decodes and carries out uniform resizing to 1024 × 576, and convex-polygon intersection and JPEG encoding are performed; all coordinates manipulation is conducted using NumPy, and Flask, along with its multi-part response generator, provides the manager and customer interfaces. Persistent saving of the detected layout to a pickle file ensures instant continuation of the session at the same slot map, while the nginx-rtmp module ensures that a temporary network delay never disrupts the state of inference.

### 3.3. Dataset

The use of any training data labelled by our custom scheme was not necessary. The YOLOv11x-OBB detector employs pre-trained DOTAv1 weights over 15 classes for overhead aerial objects, including large and small vehicles at arbitrary orientations, which readily adapts to overhead parking footage. The system was evaluated using three different aerial videos of parking lots obtained with the DJI Flip drone (Table 2): one small open parking lot (22 spaces), another medium roadside parking lot (45 spaces), and the largest one (112 spaces). Quality measures are computed based on the temporal consistency of the output without requiring annotation.

The three parking lot videos were taken from approximately 40 m in altitude with the drone’s camera looking straight downward, producing an orthographic-like perspective in which cars have rectilinear orientation and lanes are clearly visible. In these three parking lots, the first is a small open lot (22 spaces), the second a two-sided roadside lot (45 spaces), while the third is a multi-column lot (112 spaces). These three different lots were specifically selected so that we can examine the behaviour of our system as the number of spaces increases and mutual occlusion becomes harder.

All three recordings were captured at a single site (the parking facility at Multimedia University, Melaka, Malaysia). The medium lot’s central driveway casts a shadow line across nearby slots in the afternoon, and the large lot exhibited occasional truck occlusion between dense columns; both are discussed as robustness factors in Section 6. The robustness across independent sites and substantially different altitudes or lighting has not been evaluated and is discussed as a limitation in Section 7.

### 3.4. Vehicle Detection and IoU

For each detection made, the YOLOv11x-OBB detector shows a rotated four-point polygon bounding box, a main step for aerial videos in which vehicles park in bays at any orientation. Predicted vehicles having a confidence score of less than 0.4 are ignored, while the remaining polygons are converted to axis-aligned boxes for future utilisation. The IoU calculation utilises cv2.intersectConvexConvex and determines three different thresholds within the process: a 0.35 threshold for matching between slot and vehicle using a tracker; a 0.05 threshold for occupancy classification considering that vehicles can park head-in, with the bumper lying outside the bay bounds; and a 0.25 threshold for duplicate removal of synthetic slots for gap filling.

Formally, the rotated bounding boxes returned by the detector for each predicted vehicle are defined by the centre (*c_x_*, *c_y_*), width (*w*), height (*h*), and rotation angle (*θ*). Corner points are obtained by rotating the half extents offsets (Δ*x_i_*, Δ*y_i_*) ∈ {(±*w*/2, ±*h*/2)} about the centre, shown in Equation (1):

(1) $$x_{i}=c_{x}+\Delta x_{i}\cos\theta -\Delta y_{i}\sin\theta ,\;y_{i}=c_{y}+\Delta x_{i}\sin\theta +\Delta y_{i}\cos\theta$$

The resulting polygon with four points, along with each predefined polygon of the parking slot, is considered to be a planar convex polygon for which the area is calculated using the shoelace method in Equation (2), where (*x_i_*, *y_i_*) are the vertices, while the index of the vertices are modular such that the last point connects to the first one.

(2) $$A\left(P\right)=\frac{1}{2}\;\left|\sum_{i=1}^{n}\left(x_{i}y_{i+1}-x_{i+1}y_{i}\right)\right|$$

A spatial relationship measure between the detected vehicle polygon (*V*) and the parking slot polygon (*S*) is measured using the IoU ratio of their intersecting and combined areas, as shown in Equation (3). For calculating the intersecting polygon and its area, the function cv2.intersectConvexConvex is used. However, the individual area can be computed using Equation (2).

(3) $$\mathrm{IoU}\left(V,S\right)=\frac{\left|V\cap S\right|}{\left|V\right|+\left|S\right|-\left|V\cap S\right|}$$

If the maximum overlap between the parking bay and any detected polygon is greater than the occupancy threshold value (*τ_occ_*), then the parking bay is considered to be occupied, as shown in Equation (4). This low value of 0.05 caters to the head-in type of parking, where a bumper just penetrates the parking bay, but not to vehicles travelling in the adjacent lane.

(4) $$occ\left(S\right)=1\Leftrightarrow \underset{v\in V}{\max}\;IoU\left(v,S\right)\geq \tau_{occ},\;\tau_{occ}=0.05$$

In the SlotTracker (Saint Julian, Malta), if the IoU between a detection and an existing slot is higher than the stricter matching threshold (*τ_track_*) in Equation (5), then it will be considered matching. If not matching, the detection will be added to the list of candidates. However, a candidate can be marked as a confirmed slot only if it gets matched for at least *N_min_* consecutive frames, as defined in Equation (6). Moreover, there is an additional duplication threshold of 0.25 that prevents any synthetically generated slots during gap-filling overlap with pre-existing bays.

(5) $$match\left(v,S\right)=1\Leftrightarrow IoU\left(v,S\right)\geq \tau_{track},\;\tau_{track}=0.35$$

(6) $$promote\left(c\right)\Leftrightarrow h\left(c\right)\geq N_{min},\;N_{min}=5$$

### 3.5. Threshold Selection Rationale

The detection confidence threshold (0.4) follows common practice for YOLO-family deployment, balancing recall against false positives on aerial footage. The tracking-match threshold (*τ_track_* = 0.35) and the duplicate-removal threshold for gap-filled slots (0.25) were chosen to be permissive enough to re-associate a bay across the small frame-to-frame drift introduced by drone hover and encoding jitter, while still rejecting genuinely distinct bays. The occupancy threshold (*τ_occ_* = 0.05) is deliberately low to tolerate head-in parking, where a vehicle bumper legitimately protrudes past the bay boundary, without triggering on vehicles travelling in an adjacent lane. The slot-confirmation count (*N_min_* = 5 consecutive frames) balances layout stability against how quickly a newly parked vehicle is registered as a confirmed bay, and the 30-frame temporal window used corresponds to roughly one to two seconds of video, long enough to smooth single-frame detector noise while remaining responsive to genuine occupancy changes. All values were tuned by observation on a pilot recording and then held fixed across all three test lots reported in Section 6; they were not re-tuned per scenario.

### 3.6. Temporal-Consistency Quality Metrics

Since no ground-truth label exists during run time, the metrics accuracy, precision, recall, and F1 can be determined by comparing the slot labels detected per frame to the prediction obtained by the previous 29 frames using a majority vote technique. If the prediction per frame is consistent with the history, this will increase the TP or TN counts. Otherwise, FP or FN will be incremented accordingly. The calculation of such metrics starts after a minimum of 8 frames.

Specifically, the instantaneous state of each slot is determined through majority vote using a sliding window of *W* = 30 frames, as expressed in Equation (7). The smoothed result (*ŷ_i_*(*t*)) serves as the temporal ground truth of slot *i* at frame t and is equal to one if the slot is occupied in more than 50 percent of the recent frames.

(7) $${\hat{y}}_{i}\left(t\right)=1\Leftrightarrow \frac{1}{W}\sum_{k=0}^{W-1}y_{i}\left(t-k\right)\geq \frac{1}{2},\;W=30$$

By comparing the instantaneous classification results of all of the slots against the temporally smoothed results, a confusion matrix of the TP, TN, FP, and FN results is generated, in which a positive indicates the presence of an occupied bay. Using these values, Accuracy, Precision, Recall, and the F1-score are calculated by the system for every frame, except during an initial eight-frame warm-up period based on Equations (8)–(11).

(8) $$Accuracy=\frac{TP+TN}{TP+TN+FP+FN}$$

(9) $$Precision=\frac{TP}{TP+FP}$$

(10) $$Recall=\frac{TP}{TP+FN}$$

(11) $$F_{1}=\frac{2\cdot Precision\cdot Recall}{Precision+Recall}=\frac{2TP}{2TP+FP+FN}$$

It is important to note that the reference *ŷ_i_*(*t*) used above is derived from the system’s own prior predictions rather than from independently verified occupancy. The Accuracy, Precision, Recall and F1 computed by Equations (8)–(11), therefore, quantify the temporal stability/self-consistency of the system’s predictions—the degree to which a bay’s classification does not flicker between frames—rather than correctness against real-world occupancy. A prediction that is systematically incorrect but temporally stable would score highly under this measure. These figures are reported as temporal-consistency scores throughout the remainder of this paper and are distinguished from the independent detection and occupancy-classification performance evaluated against manually annotated ground truth in Section 6.

### 3.7. Independent Ground-Truth Validation Protocol

To evaluate performance independently of the system’s own temporal-consistency reference, a subset of frames from each recording was manually annotated with parking-slot locations and the per-frame ground-truth occupancy state: 31 frames for the small lot, 16 frames for the medium lot, and 4 frames for the large lot, with 51 frames in total. The small number of annotated frames, especially for the large lot, limits the precision of these estimates. Vehicle-detection performance is reported separately from occupancy-classification performance (Precision, Recall, F1 and mAP@0.5 for the former; Accuracy, Precision, Recall, and F1 for the latter), each computed against this manually annotated ground truth, so that detector errors and occupancy-logic errors can be distinguished. The detection ground truth is the annotated boxes of the slots marked occupied rather than tight boxes around each vehicle, which may lower the IoU and, therefore, the detection scores.

To isolate the contribution of each system component, four ablations are evaluated against the ground truth described above: (i) axis-aligned bounding-box detection in place of the oriented (OBB) detector to quantify the benefit of orientation-aware detection on angled aerial layouts; (ii) occupancy classification with the 30-frame temporal-majority smoothing removed, deciding occupancy from single-frame overlap alone; and (iii) automatic slot discovery with the empty-slot extrapolation routine (Section 5, Equation (12)) disabled, leaving only observed occupancy events to populate the layout; and (iv) persistent IoU-based slot tracking disabled, so that detections are re-associated to slots independently in every frame. A quantitative comparison against the published video-based YOLOv5 baseline of Ogawa et al. [14] is also reported.

## 4. Proposed Design

The proposed system is an online platform for parking-space occupation detection that uses drone photography and an implementation of the YOLO object recognition model to detect parking space occupation in real-time without employing physical sensors or predefined slot mapping. It is developed based on Flask, a web framework, and has a single background processing engine that analyses a video feed coming from either a DJI Flip drone, an existing video file, or a photo file, detecting the presence of cars, finding their parking spots, and keeping them even if the car leaves. Two separate frontends are available on the server: the first one shows the manager live footage with annotations, detection statistics, and allows for managing all parking spots, whereas the second one is designed for clients and provides them with a colour-coded SVG map of parking spots, which gets updated once per second with free slots marked as green and occupied slots marked as red without ever revealing the actual footage. Customers can access the site instantly without credentials, while managers must log in, using the public token link, and the customers’ frontend works in parallel to the manager’s session.

The architecture of the platform uses the producer–consumer approach. There is only one Detection Engine running in the background as the producer. It takes possession of the video stream, conducts YOLOv11x-OBB detection, updates the SlotTracker, conducts an occupancy evaluation and writes the annotated frame and the current layout to the shared and mutex protected buffers. The two Flask routes serve as the consumers that have no interaction with the model itself, streaming the annotated frame as a multi-part MJPEG and returning the current layout as JSON. The approach ensures that the computer-vision computation load is completely separated from the web request threads, which guarantees that any change in the number of viewers will not impact detection performance.

Theoretically, the process is through five stages: acquisition, detection, association, occupancy detection, and display. An image frame is obtained and resized. Then, the detection stage returns oriented bounding boxes, and the SlotTracker component performs slot associations with verified bays or collects the slots as potential slots. The occupancy stage will assign the status to all of the bays as either vacant or occupied and detects stray vehicles on the lane. Finally, the display layer creates a representation of the result twice: as an annotated MJPEG feed for the manager and as a simple vector map for the customer. Due to each stage writing to buffers that are controlled by locks, threading is safe even with concurrent access requests from multiple clients.

### 4.1. Login Page

**Figure 1:**
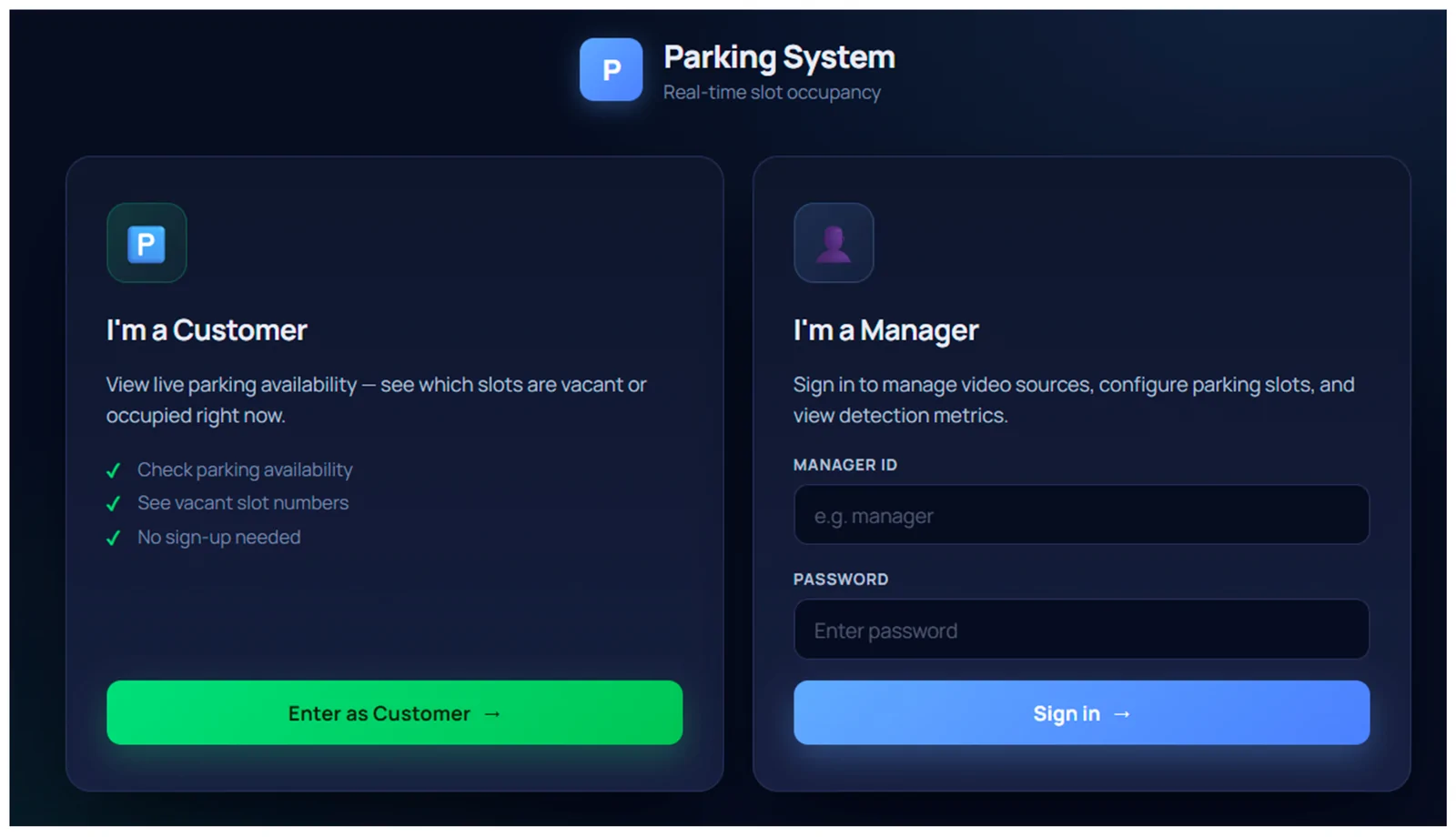
The dual-path login screen.

The login interface consists of two parallel cards. The left card, labelled “I’m a Customer”, only includes one click-button that automatically takes visitors to the map without requiring any credentials. In contrast, the right card, labelled “I’m a Manager”, needs a manager id and password to be entered to take users to the control dashboard. This two-card layout is shown in Figure 1. In Figure 1, the blue “P” icon at the top identifies the Parking System application. Left panel (Customer): the blue “P” icon denotes the customer view; the green check marks list its functions (check parking availability, see vacant slot numbers, no sign-up needed); the green “Enter as Customer →” button opens the live slot-availability view without login. Right panel (Manager): the purple user icon denotes the manager view, which requires a Manager ID and password; the blue “Sign in →” button opens the manager functions (manage video sources, configure parking slots and view detection metrics). The arrows (→) on the buttons indicate proceeding to the selected view.

### 4.2. Customer View

**Figure 2:**
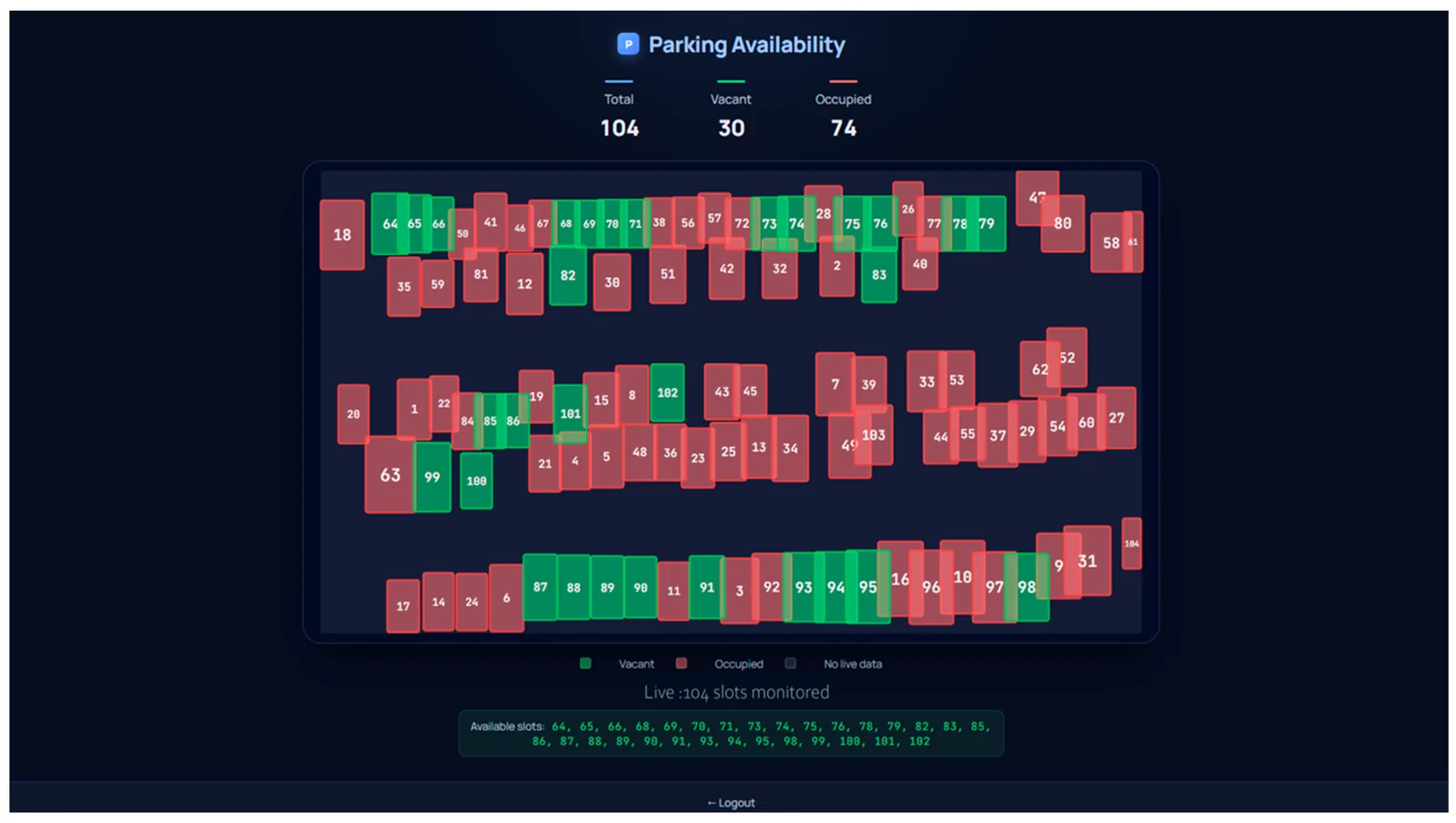
The customer’s view.

The customer’s view displays an SVG parking map whose coordinate system is defined with respect to the 1024 × 576 detection area; hence, slots can be directly drawn using coordinates without applying any transformations. Green slots are vacant slots and red slots are occupied slots, while grey slots correspond to non-active sources. The statistics bar consists of three sections that show the total numbers of slots, vacant slots, and occupied slots, while the Vacant Slots bar displays the number of all empty bays. The customer’s interface polls the/api/layout endpoint once a second, with updates occurring within two seconds. A sample screenshot is displayed in Figure 2.

### 4.3. Manager Dashboard

**Figure 3:**
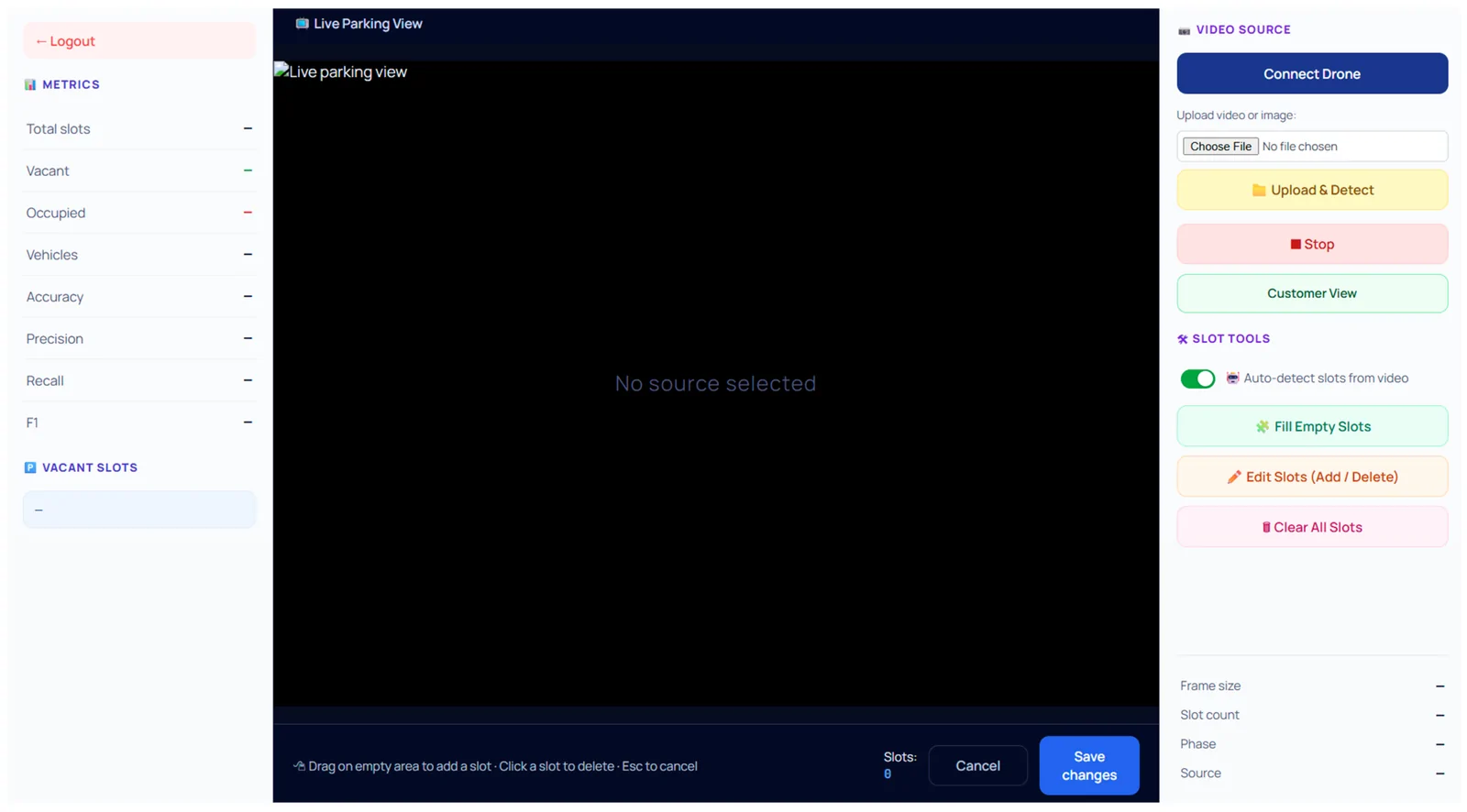
The manager’s dashboard.

The manager’s dashboard has a three-column design. The left-hand column displays eight live indicators such as Total, Vacant, Occupied, Vehicles, Accuracy, Precision, Recall, F1, and Vacant Slots list, refreshed every second. The central column feeds the live YOLO-annotated MJPEG stream, with red boxes for occupied slots, green boxes for vacant slots, and blue boxes for on-road vehicles; the stream is substituted with a canvas in the case of slot editing. The right-hand column offers video source controls, such as Connect DJI Flip, user-specified RTMP/RTSP link, file upload, and streaming cessation; slot manipulation, including an auto-detection on/off toggle, Fill Empty Slots, Edit Slots, and Clear All Slots; and diagnostics, which are the frame dimensions, number of slots, and detection status. Figure 3 shows a screenshot of the manager’s dashboard.

In Figure 3, left panel: “Metrics” shows total, vacant and occupied slots, vehicles, and accuracy, precision, recall and F1; “Vacant slots” lists the IDs of vacant slots. Centre: live parking view (black area, “No source selected” when no video is loaded); the bottom bar is the slot-editing mode, where dragging on an empty area adds a slot, clicking a slot deletes it, Esc cancels, “Save changes” stores the edits and “Cancel” discards them. Right panel: “Connect Drone” (dark blue) connects the UAV video; “Choose File” and “Upload & Detect” (yellow) run detection on an uploaded video or image; “Stop” (red) stops processing; “Customer View” (green) opens the customer map; the “Auto-detect slots from video” toggle (green when on) enables automatic slot discovery; “Fill Empty Slots” (green) fills gaps between detected slots; “Edit Slots (Add/Delete)” (orange) opens the editing mode; “Clear All Slots” (pink) removes all slots; the lower fields show frame size, slot count, processing phase and source. Dashes (–) mean no data yet.

### 4.4. “Edit Slots” Overlay and Slot Persistence

By pressing “Edit Slots”, the stream pauses and shows a canvas overlay on the current raw frame containing all the confirmed slots rendered in amber outlines. The manager can drag and create new slots and click to remove the existing ones. The conversion from screen coordinates to those of the raw frame compensates for browser letterboxing and saves the layout to slots.pkl instantly. The same configuration file is used by the command-line utilities, thus allowing for the interchangeability of layouts produced by both interfaces.

**Figure 4:**
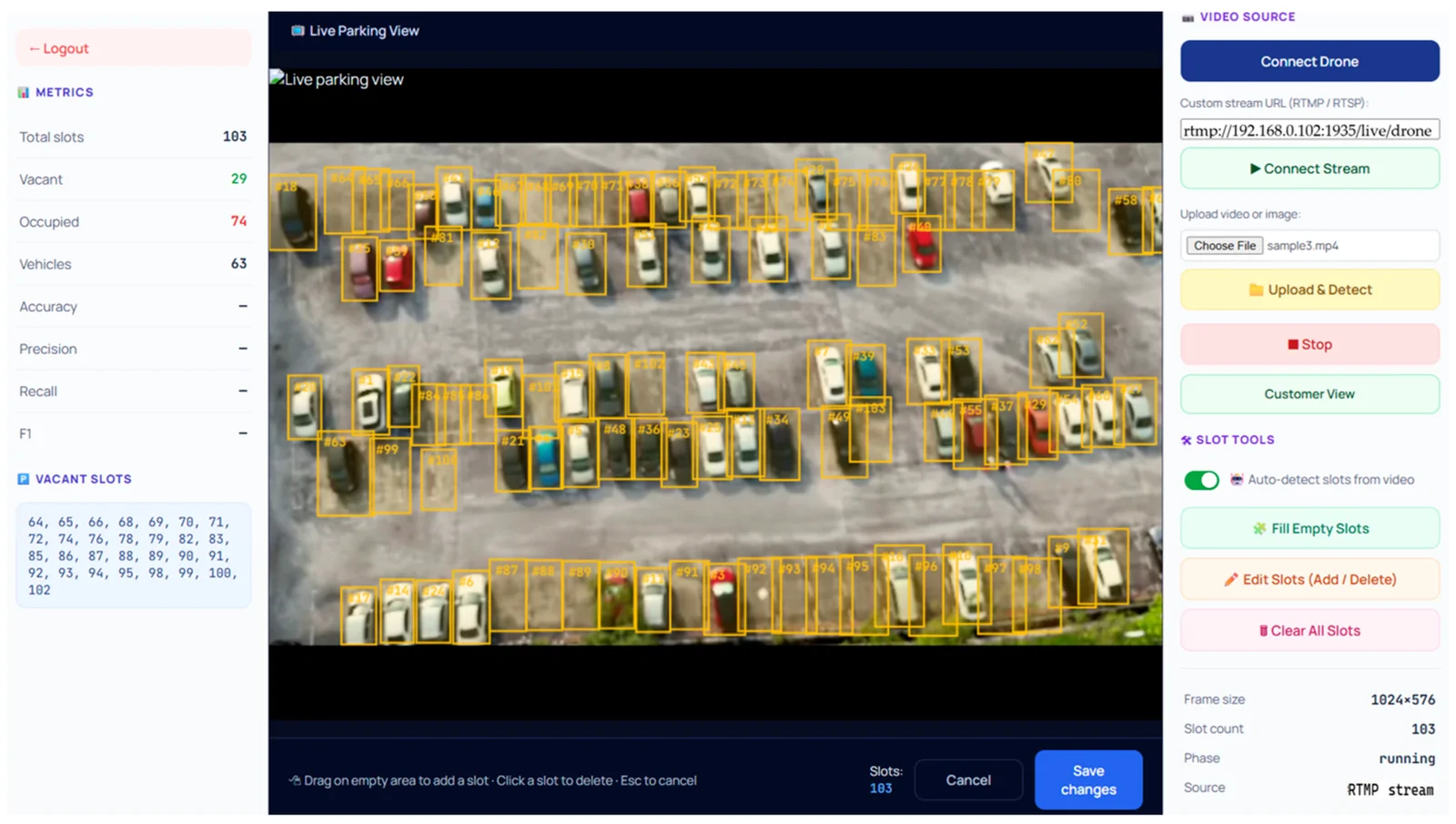
Manually editing the slots for custom RTMP stream configuration for connecting the DJI Flip drone to the smart parking detection system using the rtmp//192.168.0.102:1935/live/drone.

The yellow bounding boxes represent the detected or tracked parking slots, with each box outlining an individual vehicle or parking space candidate identified by the system. The yellow numbers are unique identification IDs assigned to individual detections or tracked slots, allowing the system to distinguish and track them across video frames. Each rectangle indicates the detected object’s approximate location and size, while overlapping boxes may occur when vehicles are closely parked or detections overlap. These visual annotations help monitor vehicle positions and support parking-slot tracking and occupancy analysis. Figure 4 shows the processing of manually editing the slots for custom RTMP stream.

## 5. Implementation

**Figure 5:**
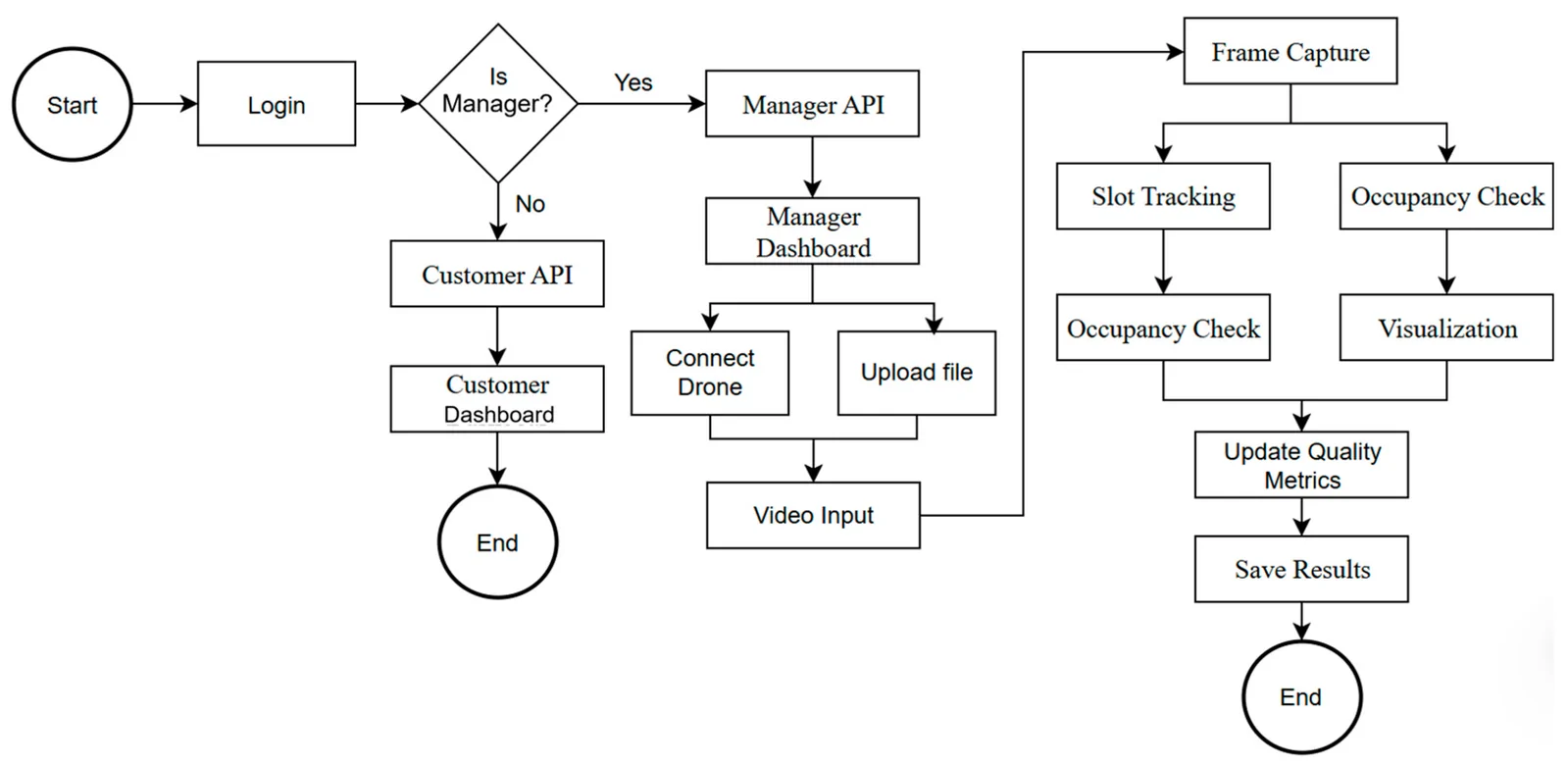
System flow diagram.

The Smart Parking Detection System operates through two user roles: manager and customer. After login, the manager uploads an aerial video/image or connects the DJI Flip via RTMP to start the detection pipeline, while customers access only the dashboard through the customer API. The DetectionEngine.set_source() loads the input source and sets up the SlotTracker using the layout from slots.pkl. Each frame is obtained with cv2.VideoCapture and resized to 1024 × 576 with a per-capture lock to avoid FFmpeg async_lock threading issues. YOLOv11x-OBB is used to analyse each frame, retain detections, with a confidence value ≥0.4, as rotated polygon detections, and then convert detections to rectangular bounding boxes. Vehicles are matched to confirmed slots using an IoU ≥ 0.35; those that are unconfirmed are considered candidates and moved into confirmed slots if they have been present in their position for ≥5 frames. The check_slots_occupancy() function evaluates each slot as either occupied (IoU ≥ 0.05) or vacant and labels all vehicles found within on-road areas outside of any slots with blue labels. “Fill Empty Slots” detects the lanes’ orientation, estimates the unit spacing with the histogram mode, and creates synthetic slots to fill gaps between lanes. Lastly, the visualize_results() annotates each slot in red/green/blue colours and streams a JPEG-encoded video with a quality of 80 through the manager API; customers can query/api/layout to retrieve an SVG-coloured parking map without watching the live video feed. Figure 5 show the system diagram for proposed system.

The process of slot extrapolation performed by clicking “Fill Empty Slots” concerns those bays that do not contain any vehicles throughout a session. Firstly, the confirmed slots are classified into lanes by the prevailing direction, and the distance between neighbouring centres within each lane forms a histogram. The most frequently observed centre-to-centre distance is adopted as the unit spacing (*u*) and insensitive to small deviations. Then, starting from the last observed bay centre (*p*_0_) and traversing the unit lane vector ($\hat{d}$), we construct a sequence of candidate centres (*p_i_*) using Equation (12) whenever a slot exists at the given location and exceeds the deduplication threshold of 0.25, and the slot is added to the set.

(12) $$P_{j}=P_{0}+ju\hat{d},\;j=1,\;2,\;\ldots ,m$$

The engine keeps track of the phase state internally. When the number of bays is less than the stable threshold, the engine remains in the discovering mode and gathers all possible candidates. However, as the engine stabilises on a particular configuration, the engine goes into the tracking mode, which tracks occupancy changes without altering anything so that new detections do not change the already existing map. A single capture lock ensures serial access to the video source to make sure the inference and reconnection threads do not invoke FFmpeg concurrently. Otherwise, an async_lock issue occurs because the RTMP video freezes. The frames with annotations are converted to JPEG with a quality of 80 and returned via a multi-part MJPEG response; the JSON configuration alone is sent to the customers.

In addition, the manager needs to transform coordinates from the fixed-size detection frame of 1024 × 576 pixels to a display area with an arbitrary width (*W_d_*) and height (*H_d_*). Preserving the aspect ratio entails stretching the frame into a letter-box format at a constant scale factor (*s*) and with offset values (*o_x_* and *o_y_*) such that a point (*X*, *Y*) in the display translates into (*x*, *y*) coordinates of the detection frame according to Equation (13). In turn, the client performs the forward mapping using an SVG viewBox that has dimensions of 1024 × 576 pixels.

(13) $$s=min\left(\frac{W_{d}}{W_{f}},\frac{H_{d}}{H_{f}}\right),\;x=\frac{X-o_{x}}{s},\;y=\frac{{Y-o}_{y}}{s}$$

## 6. Results

**Figure 6:**
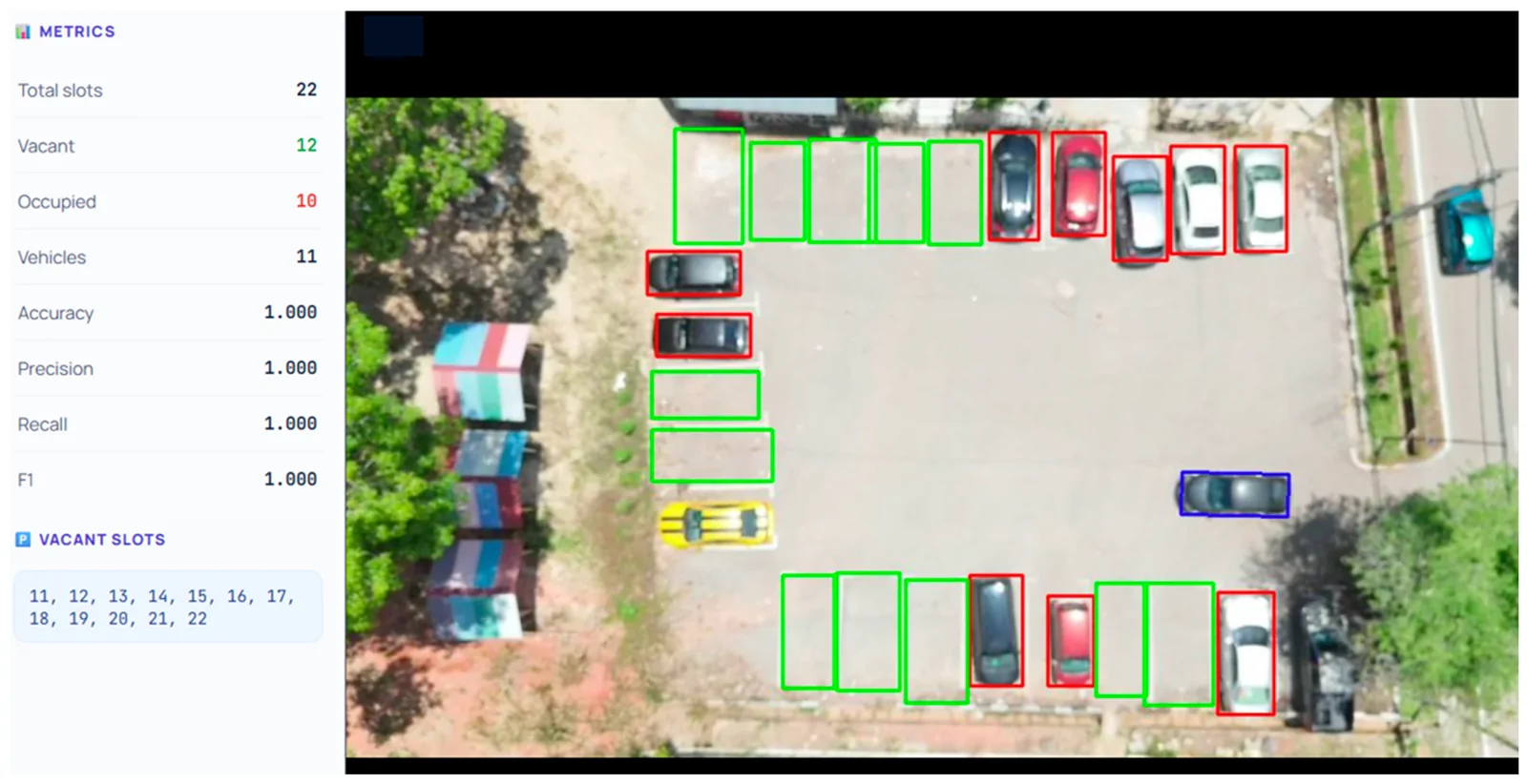
Manager view: 22 slots, 12 vacant (green box), and 10 occupied (red box), with 11 vehicles (Accuracy, Precision, Recall, and F1 = 1.000).

**Figure 7:**
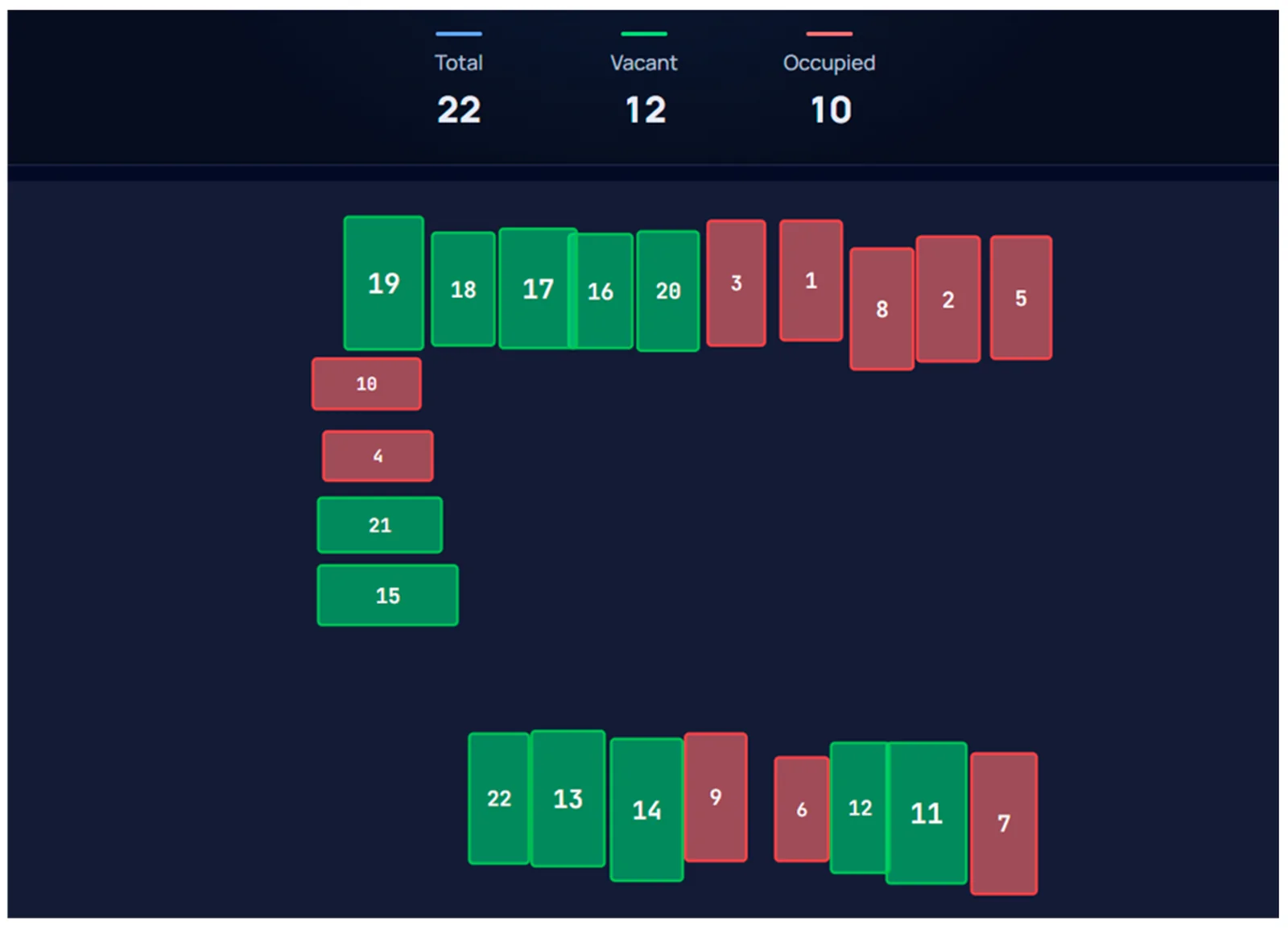
Customer map: 22 total, 12 vacant (green box), and 10 occupied (red box).

**Figure 8:**
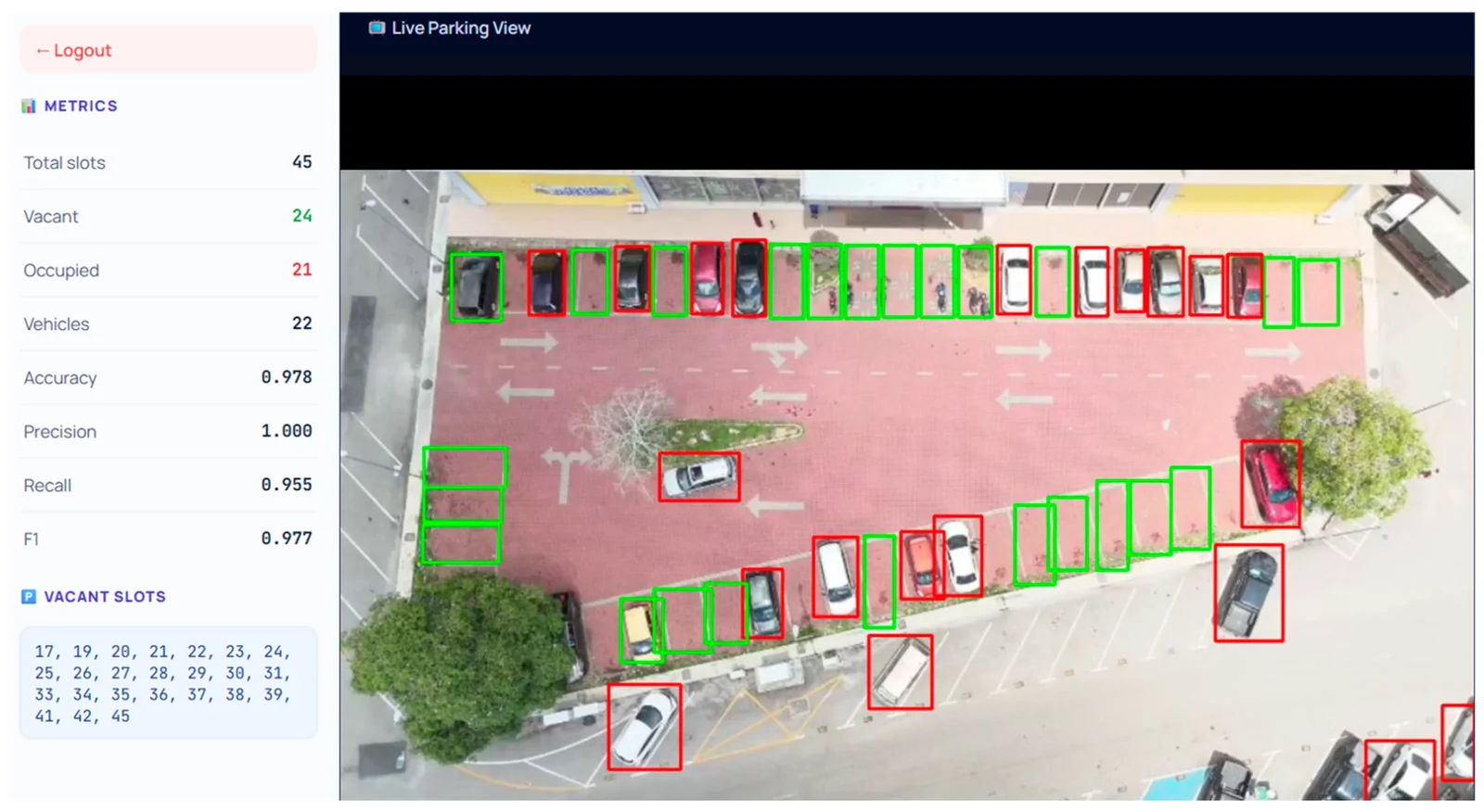
Manager view: 45 slots, 24 vacant (green box), and 21 occupied (red box), with 22 vehicles (Accuracy = 0.978, Precision = 1.000, Recall = 0.955, and F1 = 0.977).

**Figure 9:**
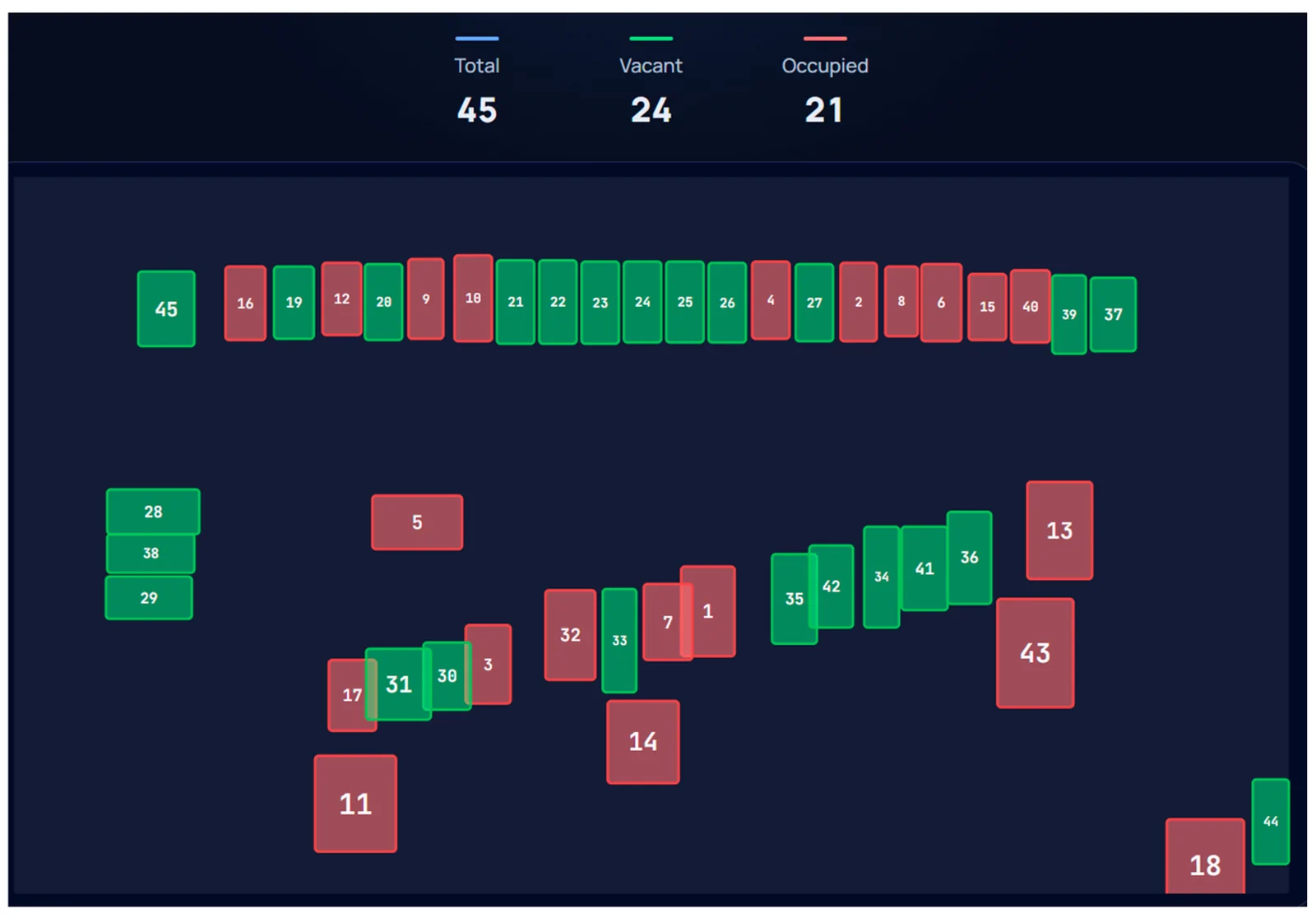
Customer map: 45 total, 24 vacant (green box), and 21 occupied (red box).

**Figure 10:**
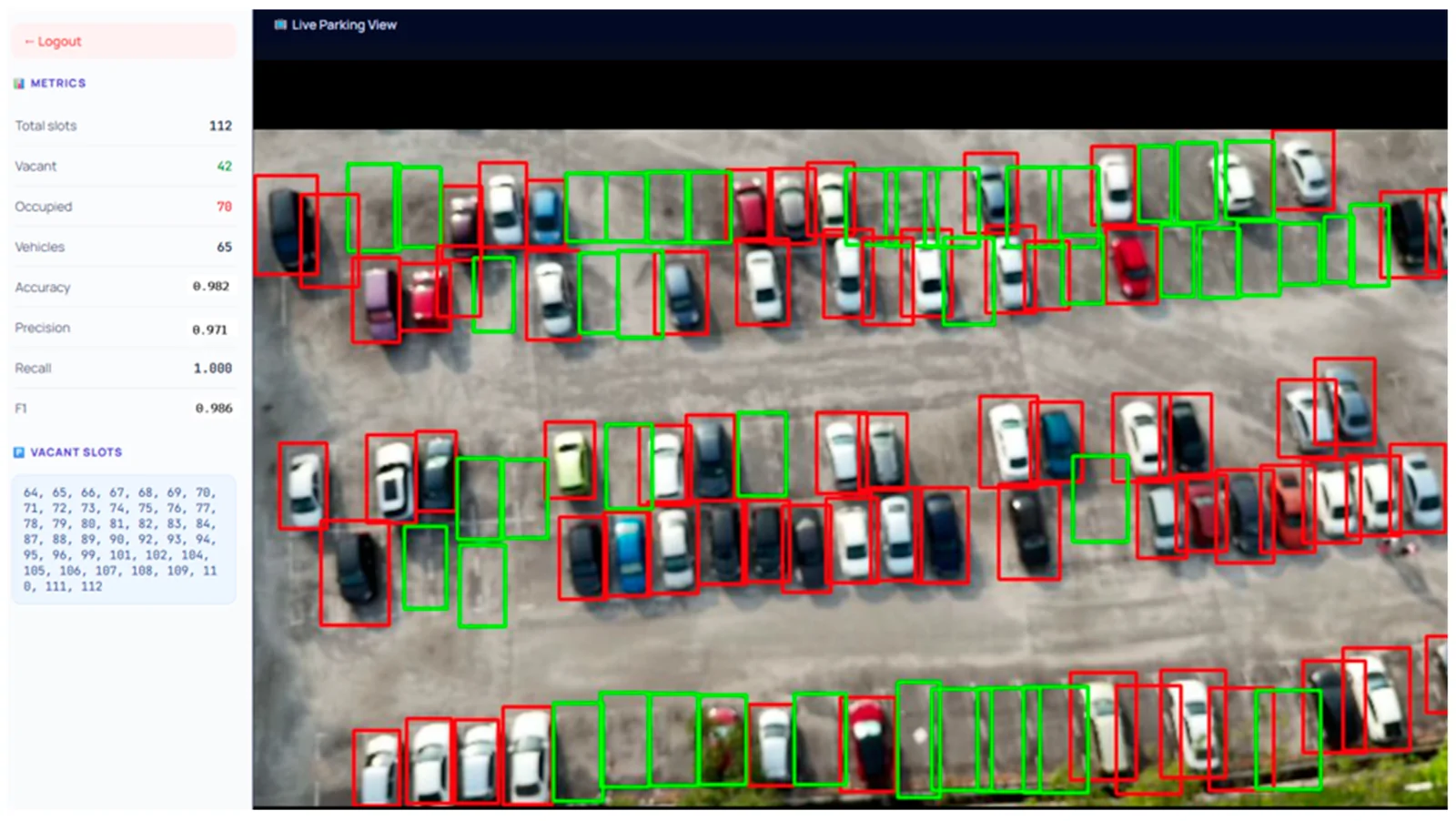
Manager view: 112 slots, 42 vacant (green box), and 70 occupied (red box). Accuracy = 0.982, Precision = 0.971, Recall = 1.000, and F1 = 0.986.

**Figure 11:**
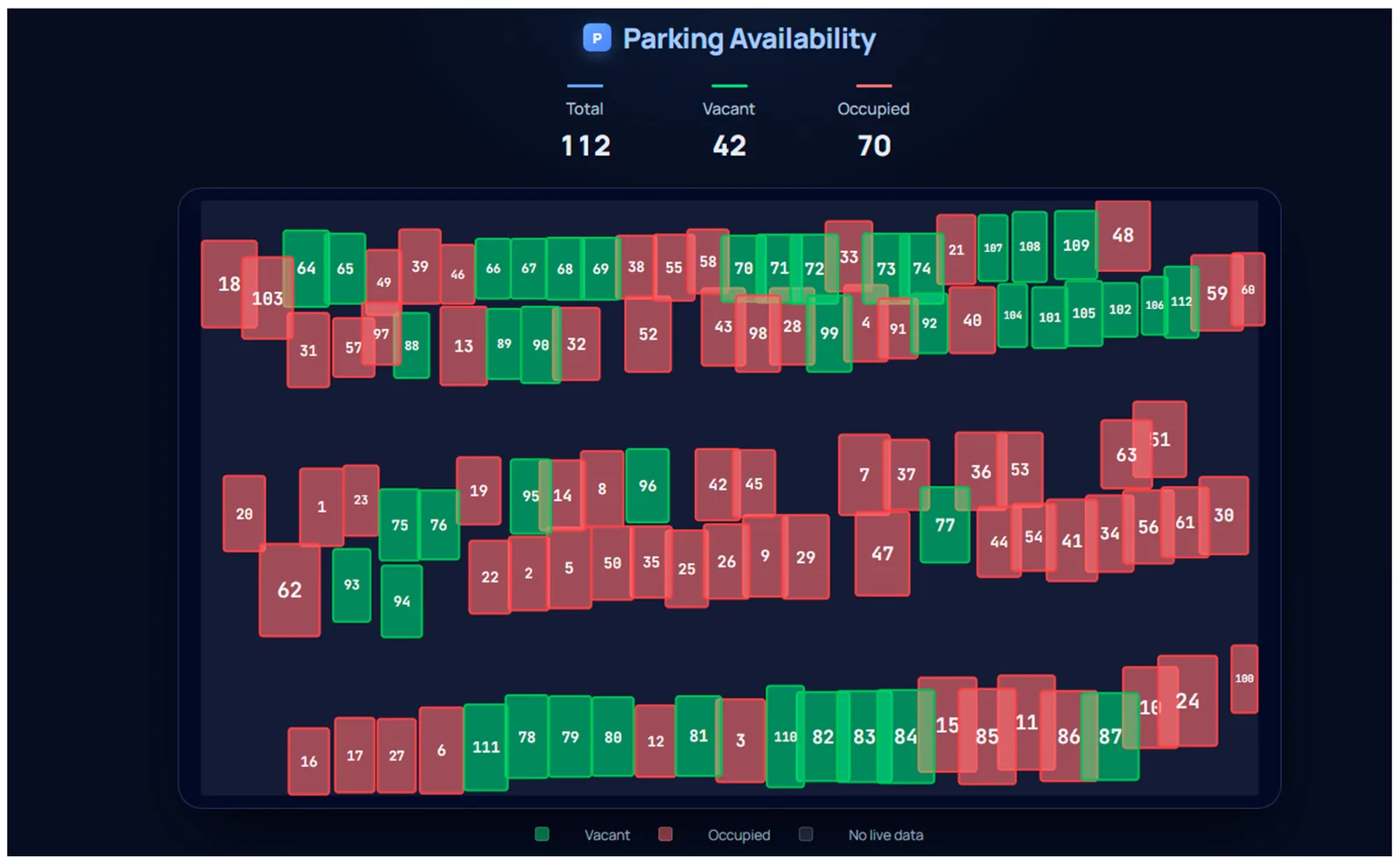
Customer map: 112 total, 42 vacant (green box), and 70 occupied (red box). Multi-row geometry faithfully reproduced.

The system was evaluated on three aerial parking lot videos recorded with the DJI Flip at an approximately 40 m altitude. Figure 6, Figure 7, Figure 8, Figure 9, Figure 10 and Figure 11 show the manager detection view and corresponding customer SVG map for each scenario. In manager view, a television system with text “Live Parking View” shown when detecting a video. A red box indicates an occupied parking slot with a vehicle inside, a green box indicates an vacant slot available for parking, and a blue box indicates a vehicle detected outside a defined slot, with the text colors matching the boxes for quick reading—green text for Vacant count, red text for Occupied count, black text for neutral metrics like Total slots, Accuracy, Precision, Recall and F1, plus a blue “VACANT SLOTS” list showing the IDs of all green-box slots. In customer view, the number inside each box is the unique ID assigned to that parking slot to identify its fixed location in the lot, while the box color shows its current status, with the top totals summarizing how many of those numbered slots are vacant, occupied and total slots. Table 3 presents the temporal-consistency detection results alongside the quality metrics for all three test scenarios.

### 6.1. Small Lot

In the images of the recording shown in Figure 6 and Figure 7, all 22 slots had been registered within about 15 s; across the annotated frames used for the independent evaluation, however, about 17% of annotated slots overall were not registered. At the time of evaluation, there were 10 filled and 12 vacant slots, with 11 cars being detected (an extra car on the travel lane as a blue polygon). All four accuracy measures were 1.000, implying total stability without flickering. Figure 6 and Figure 7 shows screenshots of the manager and customer views, respectively.

### 6.2. Medium Lot

In the images of the recording shown in Figure 8 and Figure 9, all 45 slots in the two-sided roadside setup had been registered by the algorithm at the time of testing. At the time of testing, there were 24 available and 21 taken spots, with 22 cars identified (with an extra car in the centre of the driveway). A perfect Precision of 1.000 means that there is not a single empty slot marked as occupied. A Recall of 0.955 indicates that only a few occupied slots exhibited flickering for 1 or 2 out of 30 frames of history.

### 6.3. Large Lot

In the images of the recording shown in Figure 10 and Figure 11, the system had registered all 112 slots in a crowded layout in multiple columns, the largest configuration in the test set. In total, 70 slots were occupied and 42 unoccupied at evaluation. The system detects 64 cars, fewer than the 70 occupied slots, because overlapping polygons are generated for closely parked cars that jointly occupy two bays. The Recall achieved was equal to 1.000, meaning that no occupied slot was missed throughout the entire sequence of 30 frames. The Precision was 0.971, caused by some vacant slots near the edges of the columns being occasionally covered by the neighbouring car’s bounding box. Figure 10 and Figure 11 show the manager and customer views, respectively.

Across the three different parking lot configurations, the average F1 was 0.988, and the average Accuracy was 0.987. This indicates a consistency of operation, even when increasing the lot size from 22 to 112 slots without any per-lot calibration and manual annotation. These per-lot figures describe the slots registered at the time of evaluation; slot coverage over the annotated frames is lower.

For calculation of the macro-averaged Accuracy and F1-score according to Equation (14), *m_k_* denotes the per-scenario metrics, and *S* is the number of scenarios (*S* = 3). Thus, the system achieved an average Accuracy of 0.987 and an average F1-score of 0.988.

(14) $$\bar{m}=\frac{1}{S}\sum_{k=1}^{S}m_{k}$$

The scenario-specific results also demonstrate how the system’s performance increases with the bay density. The smaller parking lot had perfect scores due to sufficient vehicle separation and absence of obstacles, resulting in flawless detection and tracking of all occupied slots without any flicker effect. With increased density, two types of errors became evident. In the medium parking lot, there was a slight decrease in Recall (0.955), since some of the occupied slots flickered in one or two of the 30 frames of history prior to stabilisation, but the Precision remained unchanged (1.000). In contrast, the large parking lot demonstrated the opposite trend, since the Recall rose back to 1.000, whereas the Precision slightly dropped to 0.971, owing to a small percentage of empty slots on the edge columns occasionally overlapping with the bounding box of the nearest vehicle. Moreover, fewer vehicles than occupied slots were detected in the densest configuration of slots because of closely parked pairs being merged into a single oriented box for two bays. This means that geometrical crowding rather than the quality of the detector should be addressed as the primary issue.

In contrast to previous computer-vision-based drone technologies, which concentrated primarily on detection and navigational functionalities [1,2,3], the suggested system goes beyond computer vision for drones to incorporate all functions associated with the parking management process, including automatic slot detection, occupancy observation, visualisation, and web interaction with users. The slot extrapolation part of Equation (12) provides even more feasibility in the form of detecting bays that are never occupied throughout the session.

To evaluate performance independently of the temporal-consistency reference used above, images were manually annotated with the slot boundaries and occupancy states. Against this ground truth, the vehicle detection and occupancy classification are reported separately in the table below (Table 4).

The independently verified detection F1 is 0.827 pooled (0.897, 0.712 and 0.871 for the small, medium and large lots) and the occupancy-classification F1 is 0.805 pooled. Both are substantially lower than the temporal-consistency scores reported in Table 3 (average F1 = 0.988). The medium lot is the weakest (detection F1 = 0.712; occupancy F1 = 0.751), and with only 51 annotated frames, with 4 for the large lot, the per-lot values are noisy. The temporal-consistency measure is, therefore, an optimistic proxy: it captures how stable the system’s own predictions are over time, while the independent evaluation additionally captures cases where the system was confidently and consistently wrong (e.g., persistent occlusion or merged bounding boxes).

Four ablated variants were evaluated against the same annotated ground truth (Section 3, Ablation Study Protocol): (i) without oriented-box detection (axis-aligned boxes only); (ii) without temporal-majority filtering; (iii) without slot-extrapolation/gap-filling; and (iv) without persistent IoU-based slot tracking, re-associating detections to slots independently for each frame instead of carrying a tracked identity forward, to isolate the contribution of the tracking itself (as distinct from the oriented-box detector evaluated in variant (i)). In addition to the occupancy-classification Accuracy, Precision, Recall and F1, Table 5 reports the Slot Recall—the fraction of the manually annotated ground-truth slots that the variant registers at all—to separate slot-discovery coverage from occupancy-classification quality.

The Accuracy, Precision, Recall and F1 are computed only over slots the variant has registered at evaluation time, so the Slot Recall—not those four metrics—is the primary indicator of slot-discovery coverage. Removing gap-filling decreases the Slot Recall from 0.833 to 0.743 (a relative loss of roughly 11%, which is consistent with the real bays left unregistered without it), while the occupancy-classification F1 on the slots it does register is essentially unaffected (0.818 vs. 0.805), confirming that gap-filling’s contribution is to the coverage rather than the per-slot classification quality. Removing oriented-box detection produces a modest drop (F1 = −0.015; Accuracy = −0.016), while removing temporal-majority filtering leaves the pooled Accuracy/F1 essentially unchanged (+0.002 F1) in this static per-evaluation-window measurement, though it still smooths the frame-to-frame flicker, which is not captured by a single pooled snapshot. Unexpectedly, removing persistent slot tracking (re-detecting and re-associating candidates independently in each frame) achieved the highest Accuracy (0.890) and F1 (0.883) of all variants, i.e., higher than the full system. We attribute this to the full system’s tracker carrying forward a slot’s prior occupancy state for a short window during state transitions, which can lag a genuine change, whereas the per-frame re-detection reacts immediately; per-frame re-detection cannot, however, maintain the persistent slot identities that slot discovery, gap-filling and the stable manager/customer map views depend on, so it is not a drop-in replacement for the full system despite the higher pooled classification score. We report this result as measured and flag it as a limitation: the present evaluation does not yet isolate tracking’s contribution to the identity persistence and discovery from its effect on the raw per-frame classification accuracy.

As detailed in Section 3.1, the system was evaluated on an NVIDIA GeForce RTX 3060 GPU (6 GB VRAM) paired with an Intel Core i7-12700H CPU. Under continuous operation, it sustained a mean of 26.3 FPS, with a mean end-to-end latency of 38 ms per frame (5 ms capture/resize, 28 ms YOLOv11x-OBB inference, 3 ms slot/occupancy logic, and 2 ms JPEG encoding and web serving). Without a GPU, the system falls back to approximately 2.1 FPS.

### 6.4. Deployment Cost Analysis

An itemised cost comparison for a representative 10,000 m^2^ (~112-bay) lot is given below, based on the hardware used in this study (camera/UAV approach) and typical geomagnetic-sensor pricing (RM 300/sensor plus gateway hardware and per-bay installation).

The camera/UAV-based approach totals RM6409, approximately 21.4% of the geomagnetic baseline (RM29,972.64) for this representative lot, in up-front purchase and installation costs. The largest driver of the difference is the per-bay sensor purchase (112 sensors), whereas the proposed system requires only a single drone/camera platform and no per-bay drilling or sealing.

Basis of the figures. All costs are stated in Malaysian ringgit (RM) on a single basis: purchase of the sensing and compute hardware plus installation labour for one 10,000 m^2^ (~112-bay) lot. The figures differ from earlier drafts of this paper. The first draft quoted USD 1409 for the camera/UAV approach versus USD 9100 for the geomagnetic baseline (15.5%); that draft mixed currencies, the geomagnetic total had been mis-calculated, and the camera-side installation-labour entry had been left empty. A later draft used an assumed unit price of RM300 per sensor (geomagnetic total = RM36,500; ratio = 12.9%); this has been replaced by a listed unit price of RM241.72 per sensor [15]. The geomagnetic total is, therefore, RM27,072.64 + RM1200 + RM1700 = RM29,972.64, and the camera/UAV total is RM1209 + RM3500 = RM4709 (negligible installation labour), i.e., 15.7% of the geomagnetic total.

Scope of the cost comparison and recurring costs. Table 6 compares the up-front purchase and installation costs and adds a recurring cost for roughly one year of continuous 24 h/day operation. The camera/UAV hardware cost of RM1209 is the DJI Store Malaysia sale price of the DJI Flip (regular price: RM1509), and the unit used here includes two batteries [16]. The flight autonomy is limited: the manufacturer’s maximum flight time is 31 min (wind-tunnel forward flight), and its maximum hover time is 28 min [17]. For continuous coverage, we assume flights of 20 min, each followed by a battery change of about 2 min, i.e., a cycle of about 22 min and about 65 flights per day. Charging two batteries from empty takes about 70 min [17], so two batteries cannot sustain continuous flying, and additional batteries would be required; we have not cost them. One operator is assumed and paid a constant RM1700 per month, the Malaysian statutory minimum wage [18], i.e., RM20,400 per year; we treat this as a fixed cost and do not model shift cover or overtime. Electricity is the only other recurring item measured. We assume (not measured) that the RTX 3060 laptop draws 150 W continuously, i.e., 1314 kWh per year, and that each of the 65 daily flights requires recharging one 22.3 Wh battery [19], i.e., about 529 kWh per year without charger losses; the total is about 1843 kWh. For the sensor baseline, we assume (not measured) a 5 W gateway, about 44 kWh per year, with battery-powered sensor nodes. At the TNB low-voltage commercial tariff of 50.68 sen/kWh plus the October 2026 automatic fuel adjustment of 3.61 sen/kWh, i.e., 54.29 sen/kWh [20], this is about RM1001 and RM24 per year; retail charges and taxes are not included. Roughly one-year totals are, therefore, about RM26,110 for the camera/UAV approach (RM4709 + RM20,400 + RM1001) and about RM29,997 for the geomagnetic baseline (RM29,972.64 + RM24), i.e., about 87%, before sensor-side servicing and battery replacement, drone repairs and additional batteries, none of which we have costed. Repair costs for DJI drones in Malaysia are quoted by one service provider at RM150 to RM300 for propeller replacement; RM300 to RM1200 for battery, motor, gimbal or light crash damage; and RM1200 to RM3000 or more for severe crash damage [21]; we do not estimate their frequency. The result depends strongly on the single-operator assumption: if round-the-clock coverage needed several staff, the camera/UAV approach would no longer be cheaper. We present these figures as an indicative comparison, and a full total-cost-of-ownership analysis, with vendor quotations for the gateway, installation and servicing and measured power draw, is left for future work.

### 6.5. Comparison Against an Existing Automatic Slot-Detection Method

Ogawa et al. [14] trained a YOLOv5 model to classify individual parking spaces as occupied or empty from a small custom dataset of 135 ground-level iPhone photographs (12.2 MP, resized to 4032 × 3024 px), labelled with the LILIN AI tool and split 90/10 into training/validation with no held-out test set. The model was trained for 50 and 300 epochs and, in addition to still images, was tested on a short video of a car entering and leaving a parking space. In comparison, the proposed system uses YOLOv11x-OBB to detect vehicles from aerial drone footage and employs a SlotTracker with IoU-based matching to determine slot occupancy, together with automatic slot discovery and empty-slot extrapolation that identifies additional parking spaces from the spatial arrangement of existing slots.

Another major difference is the system application and operating environment. The existing method targets a small, ground-level, single-site photo/video dataset with a generic occupied/empty classifier, whereas the proposed system integrates real-time aerial monitoring using a DJI Flip drone and RTMP video streaming. The proposed system further provides a complete parking management platform with separate manager and customer interfaces, where managers can configure and monitor the detection process while customers can view parking availability through a visual map. The proposed system was evaluated on three self-recorded aerial parking scenarios containing 22, 45, and 112 parking slots, achieving an occupancy-classification accuracy of 82.4% and F1-score of 0.805 against the manually annotated ground truth (internal temporal-consistency: 98.7% accuracy/98.8% F1). Therefore, while both methods address automatic parking occupancy detection, the proposed system extends the computer vision approach toward real-time drone-based parking monitoring and practical parking management.

For a quantitative, video-evaluated comparison, Table 7 reports Ogawa et al.’s [14] own published Precision and Recall for their best-performing (300-epoch) model, together with an F1-score computed from those two figures (F1 is not reported in the source paper); the proposed system’s independently evaluated figures (full-system row of Table 5, pooled over the three lots) are shown alongside for the same three metrics.

The proposed system outperforms the Ogawa et al. [14] baseline across all three metrics, most notably in the Recall (+0.09) and F1 (+0.06), although the Precision margin is small (+0.02); because the Ogawa et al. values are approximate readings from a curve, margins of this size should be read cautiously. Ogawa et al. [14] report only the approximate Precision (~0.8) and Recall (~0.7) from a Precision–Recall curve for their best (300-epoch) model and do not report the F1, which we compute here as the harmonic mean of those two approximate values; their own confusion-matrix-derived overall accuracy was substantially lower still (~41.3% at 300 epochs, ~6.5% at 50 epochs). The two studies also differ greatly in task and data: a 135-image, single-site, ground-level dataset with a 90/10 split and no held-out test set versus the present system’s purpose-built aerial pipeline with oriented-box detection, temporal tracking and gap-filling, evaluated against independently annotated ground-truth frames across three sites.

### 6.6. Discussion

First, the higher F1-score achieved by the no-tracking variant can be explained by the difference between the immediate per-frame classification and persistent occupancy tracking. In Table 5, the variant without persistent slot tracking achieves an F1-score of 0.883, compared with 0.805 for the full system. This result is reported as measured. A plausible explanation, which has not yet been verified through a dedicated experiment, is that the tracker carries a slot’s previous occupancy state forward for a short period. As a result, it may lag a genuine change in occupancy, whereas the per-frame re-detection responds immediately to changes and is, therefore, favoured by a snapshot-based accuracy measure. However, the per-frame re-detection does not maintain persistent slot identities, which are important for slot discovery, gap-filling, and the stable manager and customer map views. In addition, Table 5 does not include a metric for identity persistence. Therefore, the results show that tracking did not improve the raw per-frame classification accuracy in our measurements; they do not indicate that tracking is unnecessary. A separate evaluation using stability or identity-switch metrics would be required to quantify the benefits of persistent tracking.

Second, the relatively low Slot Recall of the full system indicates that incomplete slot coverage remains an important consideration for practical deployment. The full system achieves a Slot Recall of 0.833, meaning that approximately five out of six manually annotated slots were registered during evaluation, while roughly one in six were missed. Without gap-filling, the recall is lower at 0.743. The Accuracy, Precision, Recall, and F1-score values in Table 5 are calculated only for registered slots. Therefore, these metrics indicate how accurately the registered slots are classified, rather than how much of the parking lot is successfully covered. In a real deployment, an unregistered parking bay is excluded from the vacant and occupied counts and does not appear on the customer map. Consequently, the system may underreport the total parking capacity and the number of available spaces, even when all registered slots are classified correctly. Gap-filling improves the slot coverage from 0.743 to 0.833, but the coverage gap remains. In practice, this limitation can be partly addressed through the manager’s “Edit Slots” and “Fill Empty Slots” functions described in Section 4. A longer observation period may also allow for additional bays to be discovered. However, these effects have not yet been measured, so incomplete slot coverage remains an important limitation for fully unattended deployment.

Third, the difference between the occupancy-classification F1-score and the temporal-consistency F1-score highlights the distinction between measuring prediction correctness and measuring prediction stability. The occupancy-classification F1-score of the full system is 0.805, as reported in Table 5 and pooled across the three parking lots against the manually annotated ground truth. This is the value used to represent the system’s independently evaluated performance. In contrast, the temporal-consistency F1-score of 0.988 in Table 3 is substantially higher because it compares the system’s current predictions with its own earlier predictions. As a result, it primarily measures prediction stability and can remain high even when the predictions themselves are incorrect. The independent evaluation consists of 51 annotated frames, with 31, 16, and 4 frames from the small, medium, and large lots, respectively, covering 1995 annotated slot instances in total. Of these, 775, 688, and 532 instances come from the three respective lots. Since the large lot is represented by only four frames, its per-lot results and comparisons between lots should be interpreted with considerable caution. The pooled results are also weighed more heavily towards the small and medium lots. Furthermore, the detection evaluation is conservative because the detection ground truth is based on annotated slot boxes for occupied slots rather than tight vehicle bounding boxes. A larger independently annotated dataset, together with vehicle-level ground-truth boxes, would provide more reliable estimates of the system’s detection and occupancy performance.

Fourth, regarding generalisation and slot discovery, all recordings were captured at a single site with one drone at altitudes of 40 to 50 m in daytime conditions, so the results do not establish performance at other locations, altitudes, lighting conditions or lot layouts. The slot-discovery mechanism relies on observed occupancy and on extrapolation, which assumes regularly spaced bays, so it may be less effective in irregular layouts, and even at this site, one in six annotated slots was not registered (Slot Recall = 0.833). Validation across independent sites, altitudes and conditions is needed before the discovery mechanism can be considered effective in general.

## 7. Conclusions

The paper described a UAV-based smart parking occupancy detection system that integrates the use of YOLOv11x-OBB object detection, automatic slot discovery, temporal analysis for consistency, and a dual-purpose web application. The experiment results produced an occupancy-classification accuracy of 82.4% and F1-score of 0.805 against the manually annotated ground truth (the internal temporal-consistency Accuracy of 0.987 and F1-score of 0.988 are considerably more optimistic), with a slot-discovery coverage of 0.833, on parking lots that have varying sizes from small-sized to large open parking lots, indicating real-time operation on the tested lots, with clear room to improve the classification accuracy and slot coverage; broader scalability across sites and conditions is considered future work.

Overall, this paper offers a system-level, applied contribution rather than a new detection or classification algorithm: an automatic, annotation-free slot-discovery and tracking mechanism with row/column extrapolation that recovers never-occupied slots; a temporal-consistency measure that provides a continuous, label-free stability signal, distinct from and complementary to the independent detection/occupancy accuracy reported against the manually annotated ground truth in Section 6; and a dual-purpose web application integrating detection, tracking and visualisation into a single sensor-free pipeline. The system is designed to answer the occupancy-monitoring question—which bays are vacant—and does not provide georeferenced or GPS-level slot coordinates; it is not intended to support autonomous vehicle navigation or guidance, and the customer map is an image-plane abstraction of the observed layout rather than a georeferenced map.

Several restrictions apply to the current findings. First, the approach suffers from adverse weather conditions and poor lighting conditions. Extreme camera angles can also negatively impact the results. Secondly, the occupancy detection is based on the overlap of two planes. If vehicles are of an unusually large size or occupy closely positioned slots that merge an orientation-preserving bounding box, this may result in a false count. Thirdly, only three parking lots from one altitude level at one location were considered. Therefore, it is probable that other configurations require parameter adjustment. Fourth, the temporal-consistency scores in Table 3 are considerably higher than the independently verified figures in Section 6 (F1 = 0.988 vs. 0.805), they reflect the system’s own predictive stability rather than an independent measurement and should not be read as accuracy.

There are some directions that may be explored in future work. Multi-drone imaging with stitched images will allow for handling larger lots because it will provide wider coverage area. An infrared camera and domain-adaptation learning approach can help to deal with nighttime and adverse conditions. Multi-object tracking will preserve the integrity of slots over time when there is an extended period of occlusions. Slot learning via convolutional neural nets will reduce the necessity of regularly spaced parking slots. Finally, a cloud backend solution will enable historical and near-future prediction of occupancy in various locations. Evaluating the system across independent sites, altitudes and lighting conditions beyond the single site tested here would further substantiate the generalisation claims made in this paper.

## Figures and Tables

**Table 1:** Summary of related works.

Category	Main Methods Used	Number of Papers
UAV navigation & landing (detectors)	YOLO, YOLOv8, SSD, MobileNet	3
Fixed-camera parking occupancy (CV/DL)	Classical CV, YOLOv5/v11, slot clustering	5
UAV-based parking monitoring	Density estimation, multi-UAV, YOLOv11, VLM	6

**Table 2:** Per-lot dataset characteristics.

Lot	Duration	Frames Processed	Altitude	Occupancy Changes	Lighting
Small (22 bays)	2:30	3750	40 m	3	Clear, midday
Medium (45 bays)	3:15	4875	45 m	5	Partly cloudy, afternoon
Large (112 bays)	4:45	7125	50 m	8	Overcast, sunny day

**Table 3:** Temporal-consistency detection and quality metrics across all three test scenarios.

Scenario	Slots	Vacant	Occupied	Accuracy	Precision	Recall	F1-Score
Small lot	22	12	10	1.000	1.000	1.000	1.000
Medium lot	45	24	21	0.978	1.000	0.955	0.977
Large lot	112	42	70	0.982	0.971	1.000	0.986

**Table 4:** Evaluation against the manually annotated ground truth.

Lot	Det. P	Det. R	Det. F1	mAP@0.5	Occ. Acc.	Occ. P	Occ. R	Occ. F1
Small (22)	0.944	0.854	0.897	0.935	0.864	0.870	0.843	0.856
Medium (45)	0.688	0.738	0.712	0.706	0.784	0.715	0.791	0.751
Large (112)	0.883	0.860	0.871	0.829	0.821	0.900	0.716	0.798
Pooled (all lots)	0.836	0.817	0.827	0.839	0.824	0.819	0.792	0.805

**Table 5:** Ablation study results.

Variant	Accuracy	Precision	Recall	F1	Slot Recall
Full system (proposed)	0.824	0.819	0.792	0.805	0.833
- no OBB (axis-aligned only)	0.808	0.789	0.792	0.790	0.833
- no temporal filtering	0.825	0.820	0.794	0.807	0.833
- no gap-filling	0.838	0.845	0.792	0.818	0.743
- no tracking (per-frame re-detection)	0.890	0.880	0.886	0.883	0.815

**Table 6:** Deployment cost comparison for a 10,000 m^2^ (~112-bay) lot.

Item	Camera/UAV-Based Approach	Geomagnetic Sensor Baseline
Sensing units	1 × DJI Flip drone with two batteries: 1209	112 × sensors at 241.72 each: 27,072.64
Compute/gateway hardware	Laptop with RTX 3060: 3500	Gateway/repeater: 1200
Installation labour	1700	1700
Up-front total	6409	29,972.64
Flights per day	≈65 (20 min flight + about 2 min battery change)	—
Electricity (TNB commercial tariff)	≈1001 (≈1843 kWh)	≈24 (≈44 kWh)
One-year total	≈26,110	≈29,997
Drone repair, per event	Propeller: 150–300; battery, motor, gimbal or light crash: 300–1200; severe crash: 1200–3000+	Purchase new: 300 each

**Table 7:** Quantitative comparison against the published video-based YOLOv5 baseline of Ogawa et al. [14] (300-epoch model).

Method	Precision	Recall	F1
Ogawa et al. [14] (YOLOv5, 300-epoch, video-evaluated)	~0.80	~0.70	~0.75
Proposed system	0.819	0.792	0.805
Δ (proposed—Ogawa et al.)	+0.02	+0.09	+0.06

## Data Availability

No custom-labelled training dataset was used in this study. The evaluation videos were collected using a DJI Flip drone and are available upon reasonable request from the corresponding author.
